# Europium Metal Complex [Eu(dbm)_3_.LAP] Promotes Antinociceptive Behavior via TRPA1 Neuromodulation and an Anti‐Inflammatory Effect in Adult Zebrafish

**DOI:** 10.1002/cbdv.71141

**Published:** 2026-03-25

**Authors:** Emanuela de Lima Rebouças Borges, Antonio Wlisses da Silva, Erick Patrick Alves Moreira, Matheus Nunes da Rocha, Hélcio Silva dos Santos, Jorge Fernando Silva de Menezes, Aluísio Marques da Fonseca, Maria Izabel Florindo Guedes, Emmanuel Silva Marinho

**Affiliations:** ^1^ Biotechnology and Molecular Biology Laboratory, Health Sciences Center Itaperi Campus, State University of Ceará Fortaleza Ceará Brazil; ^2^ Postgraduate Program in Natural Sciences State University of Ceará Fortaleza Ceará Brazil; ^3^ Chemistry Course State University of Vale do Acaraú Sobral Ceará Brazil; ^4^ Teacher Training Center Federal University of Recôncavo da Bahia Amargosa Bahia Brazil; ^5^ Postgraduate Program in Energy and Environment – PGEA, Institute of Engineering and Sustainable Development University of International Integration of Afro‐Brazilian Lusophony Acarape Ceará Brazil

**Keywords:** ADMET prediction, lanthanide complex, molecular docking, molecular dynamics simulation, TRPA1 channel

## Abstract

The identification of antinociceptive compounds often relies on animal models such as zebrafish, in which TRPA1 activation induces hyperlocomotion. This study presents the first evaluation of the anti‐inflammatory and antinociceptive properties of the europium complex [Eu(dbm)_3_.LAP], derived from Lapachol, and investigates its possible interaction with the TRPA1 channel in adult zebrafish. The complex was administered intramuscularly, and its effects were compared with camphor and morphine controls. In vivo findings were integrated with in silico docking, molecular dynamics simulations, and predictive pharmacokinetic analysis. Eu(dbm)_3_.LAP (40 mg/kg, oral) was effective specifically in the inflammatory phase, and its effect was blocked by camphor, indicating TRPA1 modulation. The compound also reduced carrageenan‐induced inflammation and attenuated oxidative stress in nerve and liver tissues. In silico predictions indicated high intestinal absorption (∼100%), good blood–brain permeability (LogBB = 0.62), and low cardiotoxicity (pKi_hERG_ = 7.73). Docking revealed a binding energy of −8.1 kcal·mol^−^^1^ with stable interactions involving LYS1046, TYR1049, and LYS1052. Molecular dynamics confirmed stability over 100 ns and preservation of key hydrogen bonds, supporting the modulatory potential observed in vivo.

## Graphical abstract

The [Eu(dmb)_3_.LAP] complex was synthesized and characterized as a potent neuromodulatory and anti‐inflammatory agent. Molecular docking and ADMET analyses revealed a candidate that is permeable to the brain barrier and targets TRPA1 receptors. In vivo assays using adult zebrafish confirmed a significant reduction in inflammatory responses, highlighting this complex as a promising therapeutic strategy for neuroinflammatory disorders.

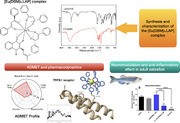

## 1 Introduction

The concept of pain was redefined in 2020 by the Taxonomy Subcommittee of the International Association for the Study of Pain (IASP) and is now defined as an unpleasant sensory and emotional experience associated with, or similar to that associated with, actual or potential tissue damage [1]. Even though they are common complaints, acute and chronic pain are often not treated satisfactorily by current therapies. This failure in treatment reflects the difficulty of developing new analgesics that are more effective, with fewer side effects and less risk of abuse than the drugs currently available [2]. In this context, the zebrafish (Danio rerio) animal model is increasingly useful for evaluating specific molecular, physiological, and behavioral phenotypes related to pain responses [3], showing promise for pharmacology and new drug discovery [4].

The discovery of antinociceptive drugs depends on animal models, such as zebrafish, where Transient Receptor Potential A1 channel (TRPA1) activation causes hyperlocomotion behavior [5]. The TRPA1 emerges as a promising new therapeutic target for nociception research. This ion channel is responsible for calcium influx into sensory neurons, functioning as a sensor of cellular damage signals, and is related to many diseases [6]. Zebrafish also emerge as a tool for discovering anti‐inflammatory drugs, as they allow real‐time assessment of cell migration mechanisms, which is crucial for understanding the inflammatory response. They also have a set of inflammatory cells, mediators, and receptors that are similar to those found in mammals, including humans [7]. Carrageenan‐induced inflammation in adult zebrafish causes abdominal edema [8], accompanied by an increase in tumor necrosis factor (TNF) levels and inducible nitric oxide synthase (iNOS) expression [9].

Metal complexes are widely used in various areas of science due to their unique electronic and stereochemical properties, and their potential for medicinal applications has been recognized and proven. Their specific characteristics—such as molecular geometries not found in organic molecules, as well as ligand exchange, redox, catalytic, and photophysical reactions—allow these compounds to interact and react with biomolecules in unique ways and through differentiated mechanisms of action, making them a promising class of drugs [10].

In light of these findings, the primary objective of the present study was to conduct the inaugural evaluation of the antinociceptive and anti‐inflammatory properties of [Eu(dbm)_3_.LAP] complex in adult zebrafish (Danio rerio). This investigation focused on elucidating its interaction and neuromodulation with the TRPA1 channel. The objective of the research is to develop novel analgesics that exhibit superior safety profiles in comparison to existing treatments.

## 2 Results and Discussion

### 2.1 Synthesis of the Complex [Eu(dbm)_3_.LAP]

The complex [Eu(dbm)_3_.LAP] manifests as a dark purple powder, indicative of the presence of naphthoquinone in the form of lapacholate ion. The complex was found to be soluble in organic solvents, such as ethyl acetate, hexane, and ethanol, and to be slightly soluble in water due to the presence of numerous organic ligands within the initial coordination sphere of the metal. The melting point of the material was measured and found to exceed 300°C, which is the maximum temperature of the apparatus used.

Table 1 summarizes the chemical composition of the Eu^3+^ complexes determined by CHO elemental analysis and the ion content in percentages found (calculated) obtained by complexometric titration with EDTA in methanol solution with the respective ligands: H_2_O (C: 65.50 (65.59); H: 4.55 (4.46); O: 14.26 (14.15); Eu^3+^: 15.09 (15.81) and LAP (C: 67.27 (67.73); H: 4.65 (4.45); O: 13.72 (13.53); Eu^3+^: 14.94 (14.28). These results agree with the formulas; [Eu(dbm)_3_.(H_2_O)_2_] and [Eu(dbm)_3_.(LAP)].

**TABLE 1:** CHO elemental analysis and the ions content in percentages found (calculated).

Metal complexes	(Elemental analysis [%]) calculated (found)			
	C	H	O	Eu^3+^
[Eu(dbm)_3_.(H_2_O)_2_]	65.50 (65.59)	4.55 (4.46)	14.26 (14.15)	15.09 (15.81)
[Eu(dbm)_3_.(LAP)]	67.27 (67.73)	67.27 (67.73)	13.72 (13.53)	14.94 (14.28)

**FIGURE 1:**
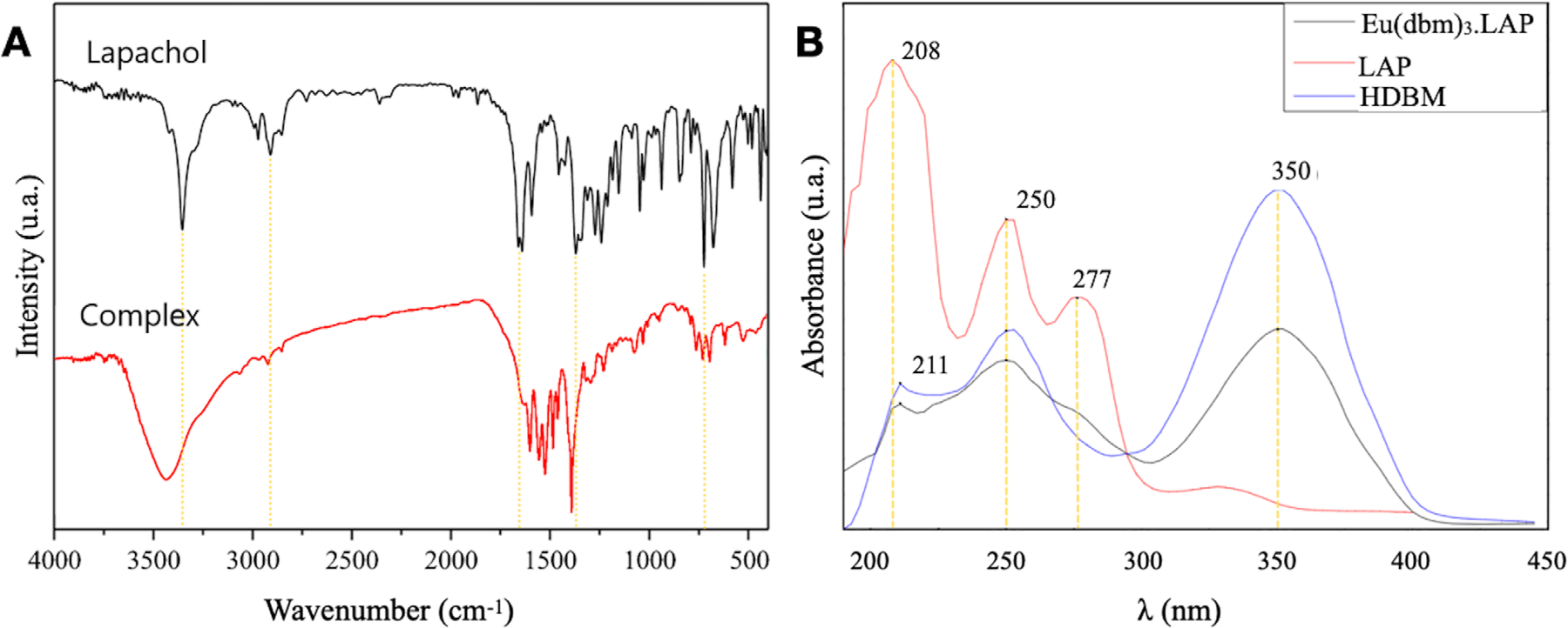
(A) Infrared (IR) spectrum of pure lapachol and [Eu(dbm)_3_.LAP] complex. (B) Complex UV‐vis spectrum of [Eu(dbm)_3_.LAP], LAP, and HDBM.

The infrared (IR) spectrum (Figure 1A) of the complex containing lapachol (LAP) purified by the new method shows an intense and broad band corresponding to the O─H group, with a maximum at 3427.84 cm^−1^, while the spectrum of pure lapachol shows a narrow band in this range, which may indicate the presence of free OH in the complex. This is significant because, according to the literature, the range from 3500 to 3100 cm^−1^ shows records of hydrogen bonding and free O─H bands. The records in the 3100–2700 cm^−1^ region are indicative of symmetric and asymmetric deformations of C─H sp2 and sp3, in relation to the branching and benzene ring of the naphthoquinone structure. A shift in the C═O bond registration was observed, with a decrease from 1640 to 1621 cm^−1^. This shift is indicative of a process of complexation with the metal center via ketone oxygen atoms. An analysis of the spectrum in the region of 1400 cm^−1^ reveals suppression in some common records of carbon chains with terminal methyl, as exemplified by methyl propene present in the lapachol molecule. This outcome suggests the development of a novel chemical species through the complexation of the ligand with the metal center. This is evidenced by the shift in the characteristic bands of the free ligand, particularly those of ketones, which are identified as coordination points, along with other bands that were suppressed.

In the Lapachol curve (Figure 1B), it is evident that the most intense peaks are located in the region between 200 and 250 nm, a range indicative of π–π* transitions. These peaks correspond to the substantial number of C═C bonds observed in the molecule's structure, extending from the bicyclic aromatic ring of naphthoquinone to the unsaturated radical. In contrast, the less intense peaks, ranging from 250 to 277 nm, are attributed to n–π* transitions, arising from free electrons in the C═O bonds of ketones. Dibenzoilmethane exhibits a more intense peak at 350 nm, indicative of n–π* transitions of free electrons in the C═O bonds of ketones, a characteristic feature of β‐diketones. Furthermore, the spectrum displays peaks with analogous intensity values at 211 and 253 nm (Table 2), which correspond to the π–π* transitions of the terminal benzene rings.

**TABLE 2:** Absorption peaks in the UV‐vis spectrum (wavelength and intensity in parentheses).

[Eu(dbm)_3_.LAP]	Lapachol	Dibenzoylmethane
211 nm (0.89)	208 nm (3.347)	211 nm (1.036)
250 nm (1.205)	250 nm (2.205)	253 nm (1.424)
352 nm (1.43)	277 nm (1.661)	352 nm (2.423)

In curve 2 of [Eu(dbm)_3_.LAP], there is an observable presence of peaks at wavelengths that correspond to those of the constituent ligands. This finding suggests a dependence of the complex's absorptivity on these ligands, a conclusion that is further substantiated by the existing literature [11]. The complex demonstrates a heightened reliance on dibenzoilmethane, attributable to its pronounced affinity for molecules of the β‐diketone group, and this is further substantiated by its preponderant composition within the complex structure (1:3:1, respectively Eu^3+^, DBM^−^, and LAP). These data corroborate the results obtained in the infrared, revealing that there is a combination of ligands with the ion, Eu^3+^.

### 2.2 In Vivo Tests

**FIGURE 2:**
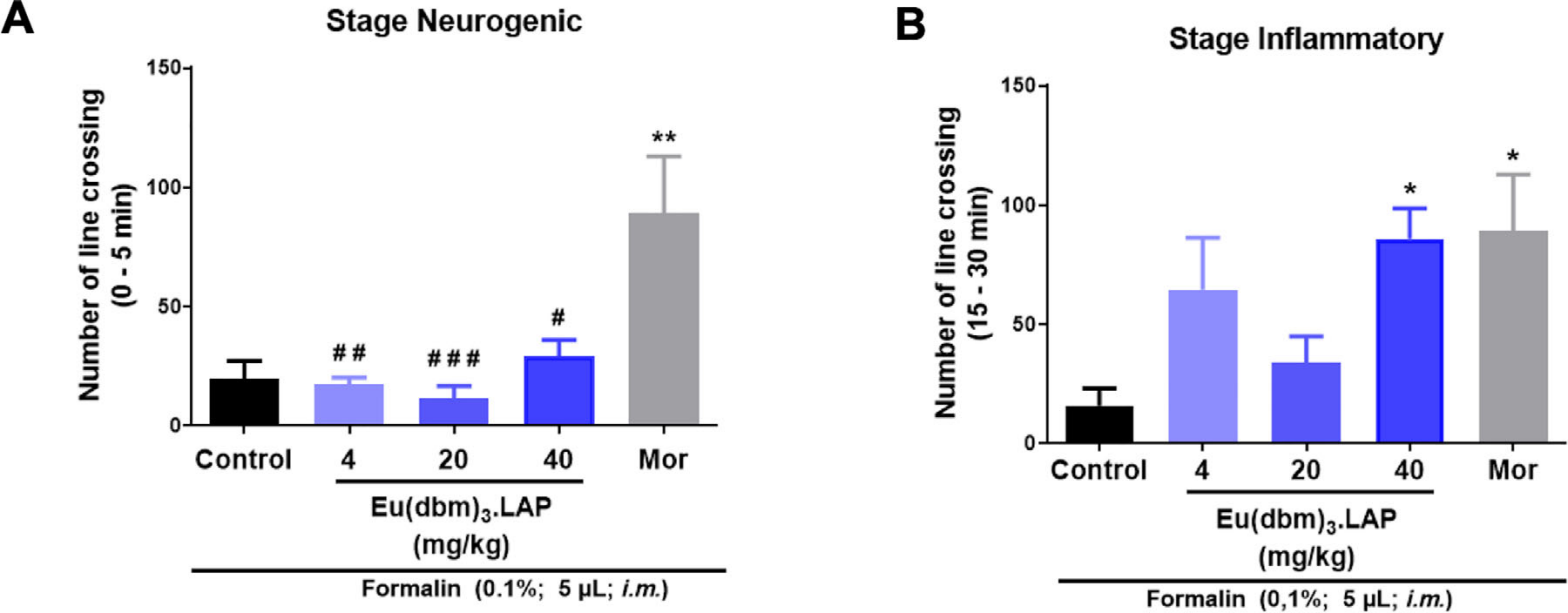
Effect of the Eu(dbm)_3_.LAP complex on formalin‐induced nociception in adult zebrafish. (A) Neurogenic phase (0–5 min). (B) Inflammatory phase (15–30 min). Data are expressed as the mean ± standard error of the mean (SEM). *n* = 6 animals per group. Statistical analysis was performed using one‐way ANOVA followed by Tukey's post hoc test. *p < 0.05, **p < 0.01 versus control group; #*p* < 0.05, ##*p* < 0.01, ###*p* < 0.001 versus morphine group. Control: vehicle (3.0% DMSO; 20 µL, p.o.); Mor: Morphine (8.0 mg/kg; 20 µL; ip).

The acute antinociceptive effect of the compound [Eu(dbm)_3_.LAP] was evaluated in the first phase (0‐5 min) of the formalin test. Statistical analysis by one‐way ANOVA, followed by Tukey's test, revealed that no dose (4, 20, or 40 mg/kg; 20 µL; p.o.) significantly reduced formalin‐induced nociception in the neurogenic phase compared to the control. In contrast, treatment with the positive control, morphine (Mor, 8.0 mg/kg; 20 µL; ip), caused a marked and significant increase in locomotor activity (**p < 0.01 vs. control), indicating a hyperlocomotion effect (Figure 2A). In the inflammatory phase (15–30 min), the 40 mg/kg dose of the compound significantly increased the number of line crossings (*p < 0.05 vs. control). The same occurred in the group treated with morphine (Mor, 8.0 mg/kg; 20 µL; ip), which exhibited a significant increase in locomotor activity (*p < 0.05 vs. control) (Figure 2B).

In adult zebrafish, pain induced by intramuscular formalin in the tail directly affects the animal's locomotor behavior, indicating a nociceptive state [12]. In the neurogenic phase (3‐5 min), formalin is believed to promote an effect on C fibers and induce the release of substance P and bradykinin. However, in the inflammatory phase, which begins 20 min after formalin application, nociception mediators such as serotonin, prostaglandin, and bradykinin are released. Opioid analgesics act as centrally acting drugs and reverse nociception in both phases, while peripherally acting compounds such as nonsteroidal anti‐inflammatory drugs (NSAIDs) and steroidal anti‐inflammatory drugs (SAIDs) act mainly in the second phase, which is mediated by inflammatory and sensitization mechanisms [13].

The metal compound [Eu(dbm)_3_.LAP] (40 mg/kg, p.o.) was effective exclusively in the inflammatory phase (15–30 min) of the formalin test, without showing significant activity in the neurogenic phase (0–5 min). This pharmacological profile suggests that the compound does not act as a direct neuronal analgesic, such as morphine (a centrally acting drug) that reversed nociception in both phases. However, the metal complex has its action intensified in the inflammatory phase, indicating possible peripheral action with an anti‐inflammatory effect.

The analgesic mechanism of action of the [Eu(dbm)_3_.LAP] complex was evaluated using a formalin model (TRPA1 receptor activator) and the TRPA1 receptor antagonist (camphor, 30.4 mg/kg; 20 µL; ip). Camphor was used in the neurogenic and inflammatory phases. A dose of 40 mg/kg (v.o.) was chosen for this study.

**FIGURE 3:**
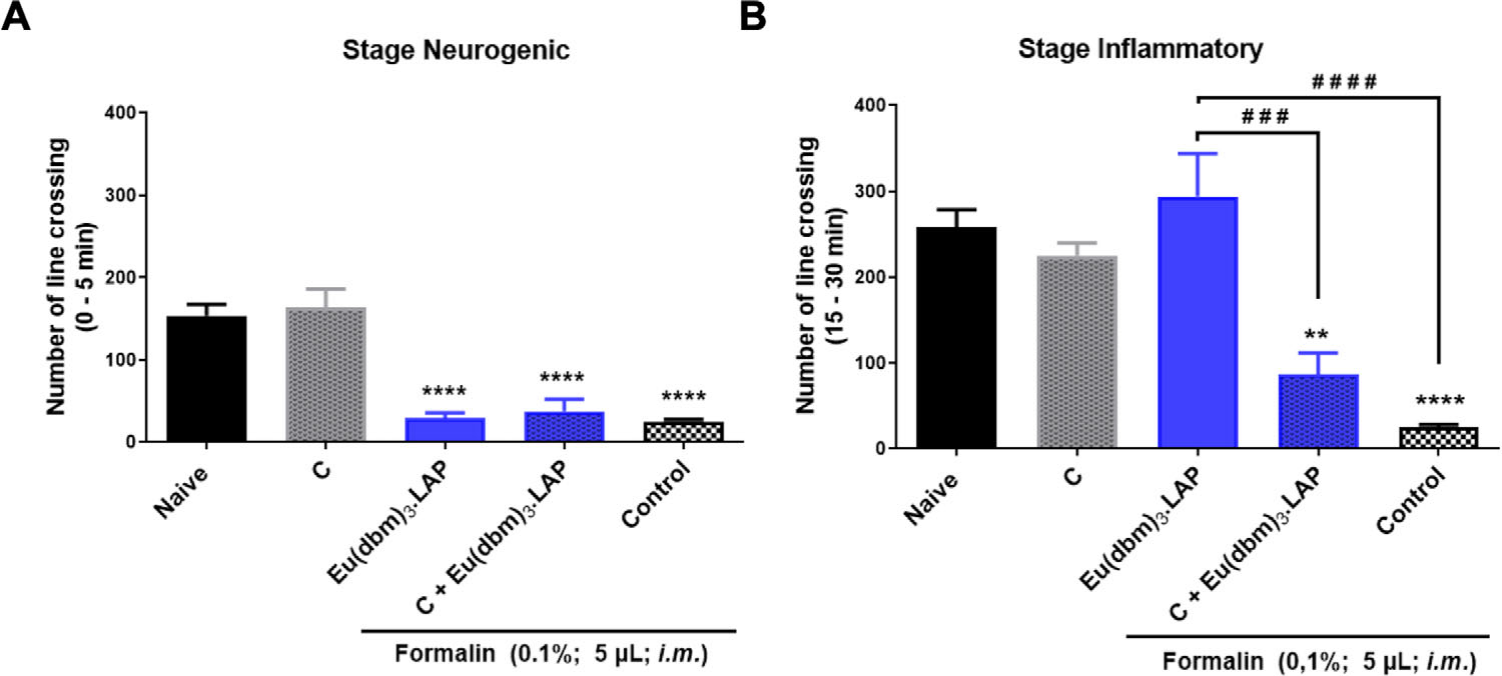
Effect of pretreatment with camphor (C) on the antinociceptive activity of the Eu(dbm)_3_.LAP complex in the formalin test in adult zebrafish. (A) Neurogenic phase (0–5 min). (B) Inflammatory phase (15–30 min). Animals received C (camphor: 30.4 mg/kg; 20 µL; ip), Eu(dbm)_3_.LAP (40 mg/kg, p.o.), or the combination C + Eu(dbm)_3_.LAP before intramuscular (im) injection of formalin (0.1%; 5 µL). The Naive group received no treatment, and the control group received a vehicle (3.0% DMSO; 20 µL, p.o.). Data are expressed as mean ± standard error of the mean (SEM), *n* = 6 per group. Statistical analysis: one‐way ANOVA followed by Tukey's post hoc test. **p < 0.01, **p < 0.0001 versus control group; ^###^ *p* < 0.001, ^####^ *p* < 0.0001 versus Eu(dbm)_3.LAP group.

In the neurogenic phase, the naive group (without treatment) exhibited basal locomotor activity (****p < 0.0001 vs. control), confirming the induction of nociceptive behavior. Camphor did not reverse the antinociceptive effect of [Eu(dbm)_3_.LAP] (40 mg/kg), maintaining a significant reduction in nociceptive activity (****p < 0.0001 vs. control) and showed no statistical difference compared to the group treated only with [Eu(dbm)_3_.LAP] (*p* > 0.05) (Figure 3A).

In the inflammatory phase, the control group reduced locomotor activity (****p < 0.0001 vs. Naive), indicating severe pain in the control group. Treatment with [Eu(dbm)_3_.LAP] (40 mg/kg) alone reversed the decrease in locomotor activity induced by formalin, promoting a marked increase in the number of line crossings (^####^ *p* < 0.0001 vs. control). The administration of camphor alone also increased locomotor activity, but to a lesser extent (***p< 0.001 vs. control). The Camphor + [Eu(dbm)_3_.LAP] (40 mg/kg) group showed a drastic and significant reduction in locomotor activity (***p < 0.001 vs. [Eu(dbm)_3_.LAP] 40 mg/kg). This locomotor suppression effect was also significant in relation to the Control group (****p < 0.0001), indicating a potential effect of the camphor complex (Figure 3B).

**FIGURE 4:**
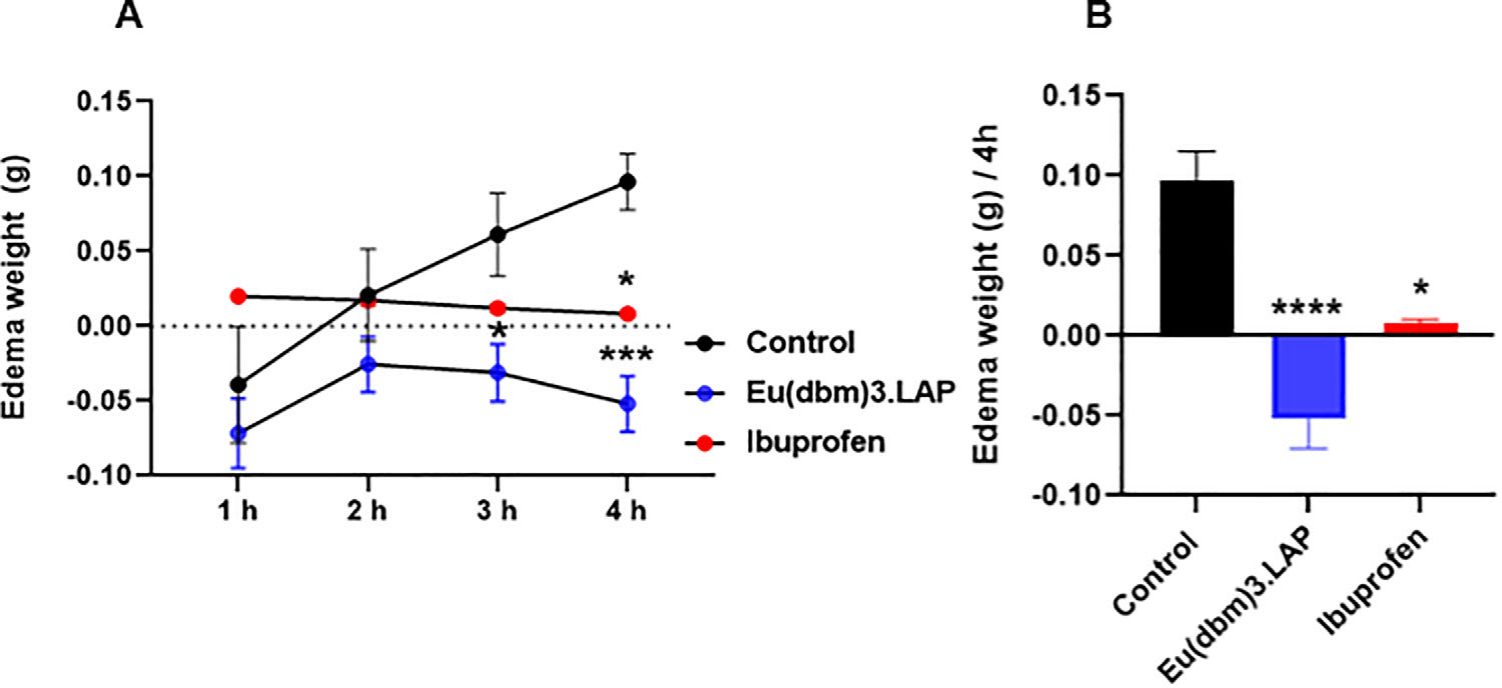
Anti‐inflammatory activity of the Eu(dbm)_3_.LAP complex in the carrageenan‐induced abdominal edema model in zebrafish. (A) Temporal evolution of edema measured hourly over 4 h. (B) Final edema weight at the 4‐h mark. Animals were pretreated with vehicle (3.0% DMSO; 20 µL, p.o.), Eu(dbm)_3_.LAP (40 mg/kg; 20 µL, p.o.), or ibuprofen (100 mg/kg; 20 µL, p.o.) before carrageenan injection. Data are expressed as mean ± standard error of the mean (SEM), *n* = 9 per group. Statistical analysis for panel (A) was performed by two‐way ANOVA followed by Tukey's post hoc test. Panel (B) was analyzed by the Kruskal–Wallis test followed by Dunn's post‐test. Symbols indicate a statistically significant difference versus the control group: * *p* < 0.05, *** *p* < 0.001, and **** *p* < 0.0001.

The data on the temporal evolution of edema, measured hourly over a period of 4 h after carrageenan injection, were analyzed using two‐way ANOVA, followed by Tukey's post hoc test for multiple comparisons (Figure 4A). The control group (dimethyl sulfoxide—DMSO 3.0%; 20 µL, p.o.) showed progressive development of edema, which became apparent from the first hour and increased in a time‐dependent manner throughout the 4‐h experiment. In contrast, treatment with the reference anti‐inflammatory drug (ibuprofen, 100 mg/kg; 20 µL, p.o.) effectively prevented edema formation at all time points evaluated, showing a statistically significant effect at the end of 4 h (*p < 0.05 vs. control). The anti‐inflammatory effect of the [Eu(dbm)_3_.LAP] group (40 mg/kg; 20 µL, p.o.) was statistically significant in comparison as early as the third hour (*p < 0.05 vs. control), reaching its maximum efficacy in the fourth hour, where the reduction in edema was highly significant (***p < 0.001 vs. control), with its effect [Eu(dbm)_3_.LAP] being visibly more pronounced than that of Ibuprofen at the end of the trial. The group treated with [Eu(dbm)_3_.LAP] showed remarkable and potent anti‐edematogenic activity.

Final weight of edema after 4 h of carrageenan‐induced abdominal edema (Figure 4B). The data were evaluated using the Kruskal–Wallis statistical test, confirming a highly significant difference (****p < 0.0001; Control vs. [Eu(dbm)_3_.LAP]. Treatment with [Eu(dbm)_3_.LAP] (40 mg/kg, 20 µL, p.o.) not only prevented edema but also promoted a net reduction in weight, resulting in a negative edema value, confirming the compound's potent anti‐edematogenic activity.

Carrageenan is a well‐established phlogistic agent that has been used for decades to induce an acute, localized inflammatory response in animal models. In zebrafish, intraperitoneal injection of carrageenan triggers a cascade of inflammatory events, inducing increased levels of tumor necrosis factors (TNF) and the expression of inducible nitric oxide synthase (iNOS) [9]. Studies have identified carrageenan as a substance that induces an acute inflammatory response in adult zebrafish and have pinpointed compounds that can reverse the inflammatory process [14, 15, 16].

Europium complex nanocomposites (Eu^+^) (Eu_2_O_3_ NRs) were used to develop injectable dressings in mouse skin models and promoted anti‐inflammatory action, inhibiting inflammatory factors (TNF‐α, IL‐6), accelerating wound healing, and increasing angiogenesis, favoring the wound healing process and skin regeneration. This study points to the participation of lanthanide ions with europium (Eu^+^) in inflammatory activity and contributes to tissue engineering and regenerative medicine [17]. The complex [Eu(dbm)_3_.LAP] (40 mg/kg; p.o.) showed anti‐inflammatory activity after induction of abdominal edema by carrageenan (Figure 4). The compound not only prevented edema but also promoted a negative edema result, confirming the complex's potent anti‐edematogenic activity. It is likely that the compound [Eu(dbm)_3_.LAP] acts in the acute phase of inflammation, modulating the animal's innate immune response, promoting a reduction in vascular permeability, inhibiting the production of inflammatory mediators, or even blocking the recruitment of immune cells to the site of injury.

**FIGURE 5:**
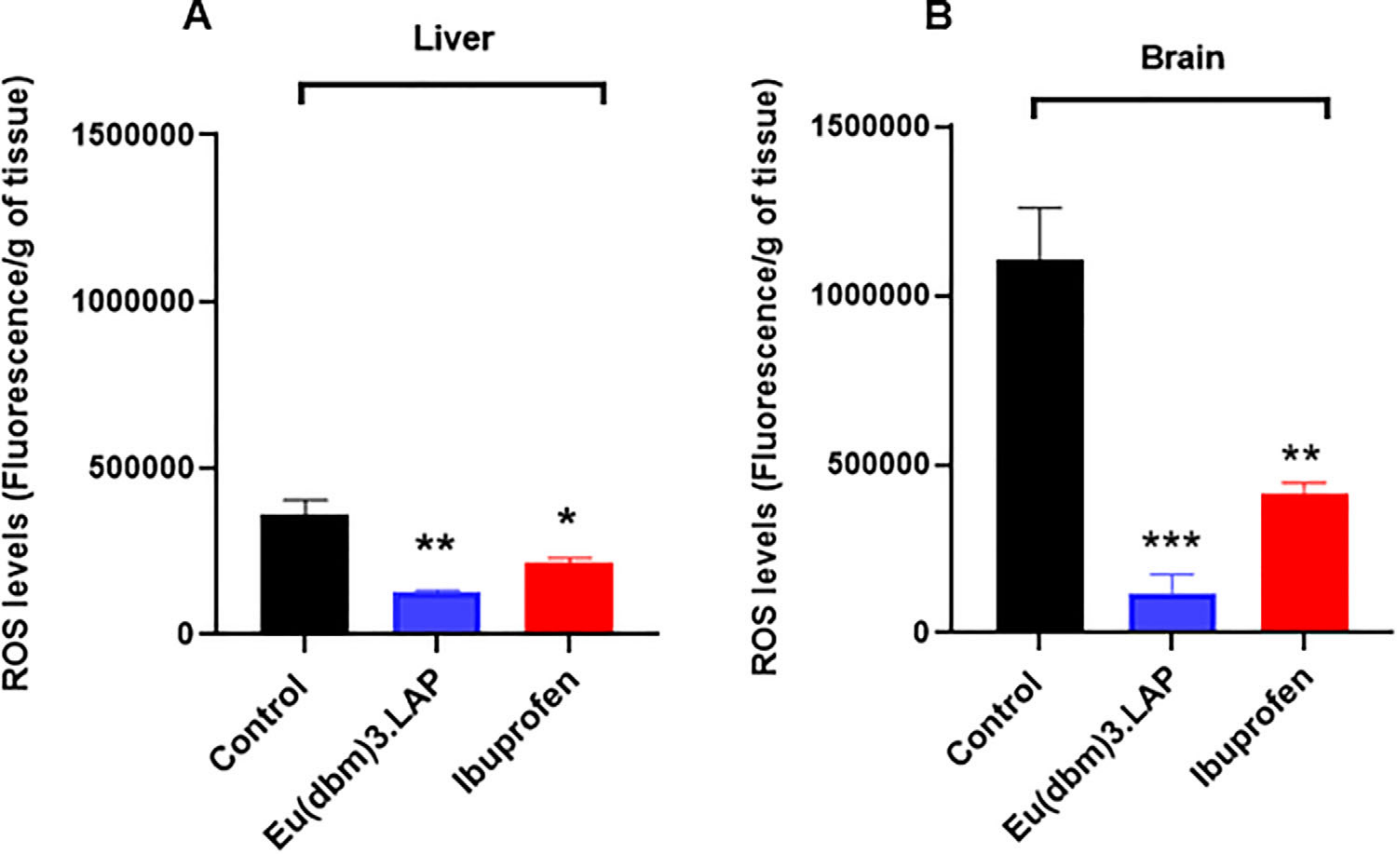
Effect of the Eu(dbm)_3_.LAP complex on oxidative stress induced by abdominal edema in adult zebrafish. (A) Brain and (B) liver. Levels of reactive oxygen species (ROS) were measured after carrageenan‐induced edema. Animals were pretreated with vehicle (3.0% DMSO; 20 µL, p.o.), Eu(dbm)_3_.LAP (40 mg/kg; 20 µL, p.o.), or Ibuprofen (100 mg/kg; 20 µL, p.o.). Data are expressed as mean ± standard error of the mean (SEM), *n* = 9 animals per group. Statistical analysis was performed using one‐way ANOVA followed by Tukey's post hoc test. Symbols indicate a statistically significant difference versus the control group: *p < 0.05, **p < 0.01, and ***p < 0.001.

To investigate whether carrageenan‐induced abdominal inflammation caused systemic damage, oxidative stress was quantified by measuring reactive oxygen species (ROS) levels in the brain (Figure 5A) and liver (Figure 5B) of zebrafish. Statistical analysis was performed using one‐way ANOVA, followed by Tukey's test. Treatment with ibuprofen significantly reduced these damage levels (*p* < 0.01 vs. control) in the brain. Even more significantly, treatment with [Eu(dbm)_3_.LAP] (40 mg/kg) promoted a drastic and highly significant reduction in oxidative stress in the brain (***p < 0.001), demonstrating a potent neuroprotective effect that was superior to that shown in Figure 5A.

Both treatments were effective in protecting liver tissue, with ibuprofen (*p < 0.05 vs. control) and [Eu(dbm)_3_.LAP] (**p < 0.01 vs. control) significantly reducing oxidative stress. Again, [Eu(dbm)_3_.LAP] demonstrated more pronounced protection in the liver (Figure 5B). Reactive oxygen species (ROS) consist of a wide variety of oxidizing molecules with very different biological properties and functions, ranging from signaling to causing cellular damage [18]. Inflammation and oxidative stress are related pathophysiological events. In addition to promoting the release of inflammatory mediators, inflammatory cells also promote the release of reactive oxygen species at the site of inflammation, leading to excessive oxidative stress. In addition, reactive oxygen species can initiate a cascade of intracellular signaling that increases pro‐inflammatory gene expression [19].

In rats, increased oxidative stress can be observed through the induction of carrageenan edema, promoting the activation of immune system cells through the release of pro‐inflammatory mediators and cytokines [20]. In adult zebrafish, ROS damage has also been investigated after carrageenan‐induced abdominal edema [8, 15, 16]. In this context, [Eu(dbm)_3_.LAP] (40 mg/kg) promoted a reduction in reactive oxygen species in the brain and liver, with its greatest protection being in the nervous system. These results suggest neuroprotective and hepatoprotective effects of the [Eu(dbm)_3_.LAP] complex (Figure 5A,B).

The efficacy of the complex was found to be exclusive to the inflammatory phase (second phase) of the formalin test. Drugs that act in this phase generally have a peripheral action, such as NSAID drugs [21]. In addition, the significant anti‐edematogenic effect observed in the carrageenan model lends further support to the action on peripheral inflammatory mediators.

### 2.3 Molecular Docking Simulations

**FIGURE 6:**
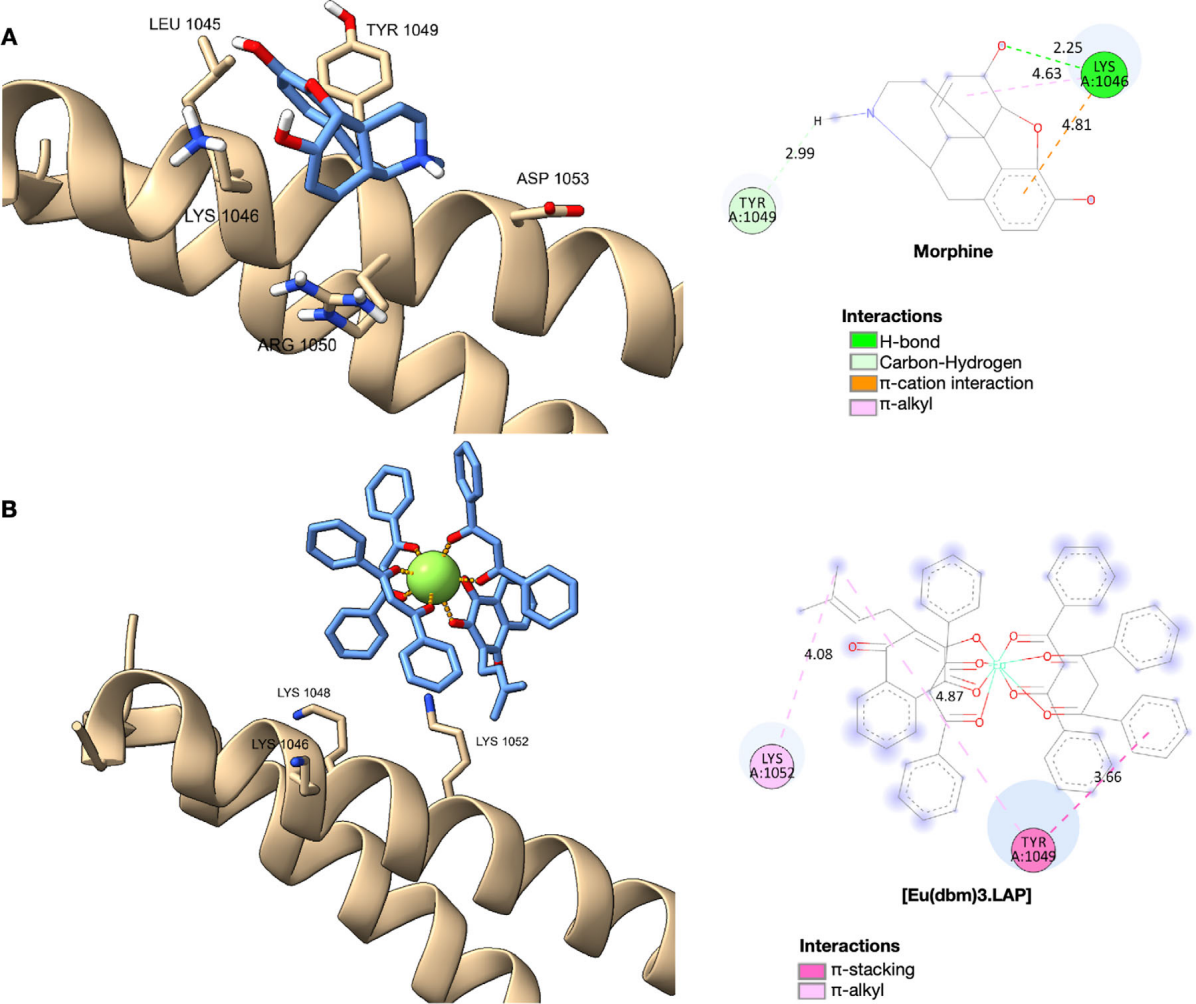
The ligand‐protein interactions of morphine (A) and [Eu(dbm)_3_.LAP] (B) with the active site of TRPA1.

In the docking analyses (see Figure 6 and Table 3), morphine adopted a well‐defined pose near the helix region that contains the residues Leu1045, Lys1046, Tyr1049, Asp1053, and Arg1050. There were short hydrogen or polar interactions with Tyr1049 (∼2.99 Å) and Lys1046 (∼2.25 Å). These interactions suggest that morphine fits into a relatively restricted pocket in which steric complementarity and polarization favor a specific ligand orientation. This pattern is consistent with the mechanism by which small molecules modulate proteins, whereby protonated groups (e.g., the morphine amine at physiological pH) form strong interactions with acidic or polar residues [22, 23, 24].

**TABLE 3:** Scoring results for the protein fit calculations used as a target for TRPA1.

Ligands	ΔG (kcal/mol)	RMSD (Å)
**Native (morphine)** ^a^	−7.4	1.8
**[Eu(dbm)_3_.LAP]**	−8.1	2.0

^a^ TRPA1 inhibitor.

Conversely, the europium complex formed extensive bonds with the InsP_6_ binding domain of TRPA1, establishing multiple contacts with the basic residues Lys1046, Lys1048, Arg1050, and Lys1052. These interactions span distances ranging from approximately 3.6 to 4.9 Å, forming a multidentate network of electrostatic interactions and relatively weak or intermediate hydrogen bonds. This could potentially compete with natural InsP_6_ or alter the conformation of this regulatory domain. Lanthanides such as Eu^3^^+^ are known for their strong polarization and for forming stable coordination bonds with oxygenated ligands in proteins. However, many conventional docking methods do not accurately predict these interactions [22, 23].

The location of the residues Lys1046, Lys1048, Arg1050, and Lys1052 within the InsP6 domain is of particular significance. As revealed by cryo‐electron microscopy, this domain plays a central role in conformational stabilization and channel gating, possibly through InsP_6_ binding, which helps to maintain inactive states or modulate conformational transitions [25, 26]. The interactions detected for the europium complex suggest that it may act as an allosteric modulator or even an antagonist by disrupting or replacing InsP6 interactions. In contrast, morphine appears less capable of interfering in such a comprehensive manner.

However, the largest network of contacts in the metal complex does not guarantee superior functional affinity. Many factors affect the stability of the bond in vivo, including desolvation, InsP6 domain dynamics, competition with endogenous InsP6, and the ligand's ability to reach the domain. Additionally, parameterizing metals in traditional docking and treating Eu^3^^+^ coordination geometry can introduce artifacts [22, 27]. The models used here clearly have limitations, including the lack of explicit coordination of water, induced polarization, and complete protein flexibility.

These results have implications for designing TRPA1 modulators. Due to its specific pose, morphine can serve as a “platform molecule” for deriving compounds with affinity for the InsP6 domain, thereby minimizing off‐target activities. Although the europium complex is promising in terms of luminescent signal or “competitive block” of the InsP6 domain, questions remain regarding permeability, toxicity, cellular delivery, and especially functional specificity. For example, it is unclear whether disturbances in the InsP6 domain result in channel activation or inhibition or if the cell's recovery/regulation cycle compensates for these interferences.

### 2.4 Molecular Dynamics Simulations

A thermodynamic system involving solutes and solvents can be conceptualized as a complex comprising protein‐ligand‐solvent‐ion interactions. Various intermolecular forces govern the interactions within this system, accompanied by heat exchanges between molecules and ions. According to the principles of thermodynamics, the relationship between these molecular entities and heat transfer mechanisms is intrinsically linked to multiple transformations and redistributions of energy [28, 29, 30].

In this context, MD simulations provide a powerful tool for investigating the behavior of protein‐ligand complexes at the atomic level. Using the NAMD software package, simulations were performed to analyze global conformational changes, monitor the stability of protein structures in different conformations, and extract critical insights into the interaction mechanisms governing these complexes [31, 32]. The resulting data offer a comprehensive understanding of the thermodynamic and kinetic properties of these molecular systems, shedding light on the underlying processes that dictate the conformational landscapes and dynamic stability of protein‐ligand assemblies.

**FIGURE 7:**
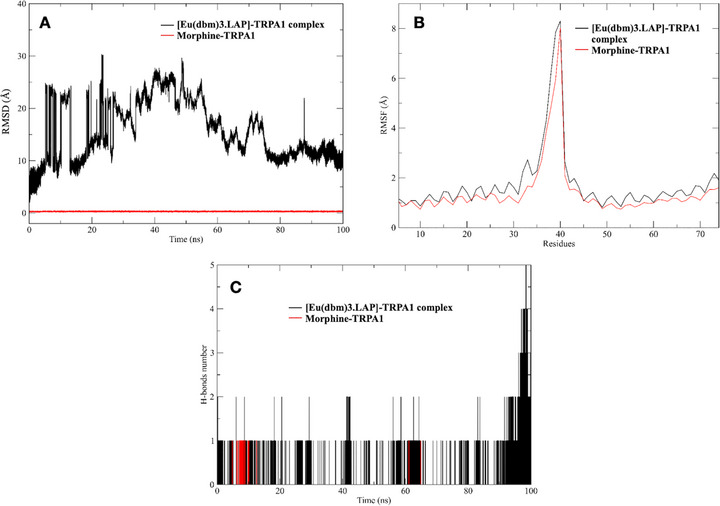
(A) Mean square deviations (RMSD) of the protein/ligand over 100 ns of simulation for TRPA1. (B) Mean square fluctuations per residue (RMSF) in protein–ligand complexes. A reduction in flexibility is observed in loop regions near the active site in the presence of both ligands. (C) Number of protein–ligand hydrogen bonds over 100 ns. The [Eu(DBM)_3·_LAP]/TRPA1 complex exhibited the highest number of simultaneous interactions, while morphine exhibited fewer H‐bonds.

Immediately after molecular docking, the ligands with the best binding energies were selected to perform the molecular dynamics study, together with the reference ligand for comparison, morphine, according to the catalytic site of the protein (Figure 7). It was observed that the average RMSD values for all 100 ns simulations in the production stages were around 0.2 to 0.3 Å for morphine, while the complex with europium, [Eu(dbm)_3_.LAP], showed immense instability, reaching around 30 Å at 23 ns, which only stabilized at the end of the simulation at 94 ns. During the production phase (100 ns), RMSD analysis revealed marked differences between the ligands in relation to the protein studied. This result indicates that the native ligand promoted a stronger and more consistent interaction with the protein, unlike [Eu(dbm)_3_.LAP], which exhibited greater deviation and fluctuation, suggesting greater flexibility or conformational adaptations.

The RMSF of the system was performed to understand the displacement and stability of each protein residue in the 100 ns simulation trajectory. Figure 7A,B superimpose the main interactions of the main complexes studied. Thus, it can be concluded that there were significant conformational changes in the protein‐ligand complexes during the simulation times [33, 34]. The two complexes used for the study, formed between ligands and the protein, presented values greater than 2.0 Å in the residual intervals 33‐39 (equivalent to His481 and His487). These fluctuations showed compromised stability of the structures in aqueous solution, evidenced in the RMSD intervals. These conformations obtained in the MD simulations for both proteins were complexed with various ligands by docking techniques, generating important information about the binding modes of small molecules in different states of enzyme folding. RMSF analysis also allowed us to evaluate residual mobility and map the regions most impacted by the presence of each ligand. Therefore, in both complexes, the ligands present reduced fluctuation in loop regions near the binding site, suggesting a local stabilizing effect.

The number of hydrogen bonds is essential to verify whether a complex has reached stability in a dynamic system [35]. After 100 ns, it was possible to verify the hydrogen bonds formed between the proteins and their respective simulated ligands in long stages of molecular dynamics production. The temporal profile of hydrogen bonds (Figure 7) revealed striking differences between the systems. The europium‐containing complex maintained a more extensive and persistent network of hydrogen bonds throughout virtually the entire 100 ns simulation compared to the native ligand. While the morphine/TRPA1 system mostly showed a single stable hydrophilic interaction, the europium system exhibited fluctuations of two to five simultaneous bonds, especially in the last 20 ns of simulation, suggesting reorganization and progressive strengthening of the interaction network at the active site of TRPA1. In quantitative terms, the average number of H‐bonds for the europium complex was approximately double that observed for morphine, reflecting greater complementarity of the metal ligand with polar residues of the receptor.

This difference in dynamic behavior was also reflected in the estimated entropic components. The formation of a more restricted complex, with a greater number of hydrogen bonds, implies a greater loss of translational, rotational, and vibrational degrees of freedom of the free molecules, directly impacting the entropy variation. The calculation of normal modes indicated that, although the entropic term is unfavorable, it is compensated by the additional enthalpic interactions provided by the europium complex, resulting in global free energy comparable to that of morphine.

The results obtained indicate that, even though it has slightly less favorable free energy than morphine, the [Eu(DBM)_3_·LAP]/TRPA1 complex forms a more cooperative and stable system over time. The progressive increase in the number of hydrogen bonds suggests not only strong initial interaction, but also a process of conformational adaptation that leads to stabilization of the metal complex within the receptor cavity. This plasticity may be associated with the polydentate character of the ligand and the coordination ability of the europium ion, which, when accommodated in the active site, promotes reorganization of the protein microenvironment. Furthermore, the extensive network of hydrophilic interactions observed for the europium complex may contribute to longer residence time and lower spontaneous dissociation of the ligand, which are desirable characteristics from a pharmacological point of view [20].

### 2.5 MM/GBSA Calculations

The estimation of free binding energies for macromolecular systems, such as protein–ligand or metal–ligand complexes, is a crucial step in assessing intermolecular stability and affinity. A widely validated approach for this purpose is the combination of molecular mechanics with the generalized born and surface area continuum (MM/GBSA) implicit solvation models [36]. This method allows the total free energy of the complex to be estimated by integrating the internal energy terms (van der Waals, electrostatic, and binding interactions) with polar and nonpolar solvation contributions, providing a more realistic picture of the association process.

In the present study, the MolAICal package was used as a computational tool to quickly and reliably estimate free binding energies from trajectories obtained by molecular dynamics. This “three‐trajectory” approach allows for a more detailed description of the free state and the complex state, incorporating conformational variations and solvent effects.

The results indicated that the morphine/TRPA1 complex had the most favorable free energy (−9.81 kcal·mol^−^^1^), marginally surpassing the [Eu(dbm)_3·_LAP]/TRPA1 complex, whose estimated free energy was −9.38 kcal·mol^−^^1^ (Table 4). Despite the relatively small numerical difference, both systems exhibit energy profiles consistent with high‐affinity interactions, highlighting the competitive potential of the europium complex over morphine at the active site of TRPA1.

**TABLE 4:** Free energy estimation data of ligands against TRPA1.

Complex	∆*E*_ele_ + ∆*G*_sol_ (kcal/mol)	∆*E*_vdw_ (kcal/mol)	∆*G*_bind_ (kcal/mol)	Standard deviation
**[Eu(dbm)_3_.LAP]**/**TRPA1**	6.95	−16.33	−9.38	±0.0134
**Morphine**/**TRPA1**	4.31	−14.12	−9.81	±0.0541

The entropic contribution, often neglected in simplified analyses, plays a fundamental role in the stability of complexes. The formation of macromolecular adducts involves a significant loss of translational, rotational, and vibrational degrees of freedom of isolated molecules, resulting in an unfavorable entropic term [37, 38]. This variation can be quantified by normal mode calculations, which provide an approximation of the internal fluctuations and conformational restriction associated with the binding process.

Thus, for a macromolecular complex involving a target and a ligand, the total interaction energy must be estimated by integrating the enthalpic and entropic components [28, 37, 39]. This approach provides a more comprehensive view of the molecular recognition mechanism, being especially relevant for systems containing metal ions, such as the [Eu(DBM)_3·_LAP] complex, in which electrostatic and coordination effects contribute decisively to the free energy of association.

### 2.6 Multivariate ADMET Descriptors

**FIGURE 8:**
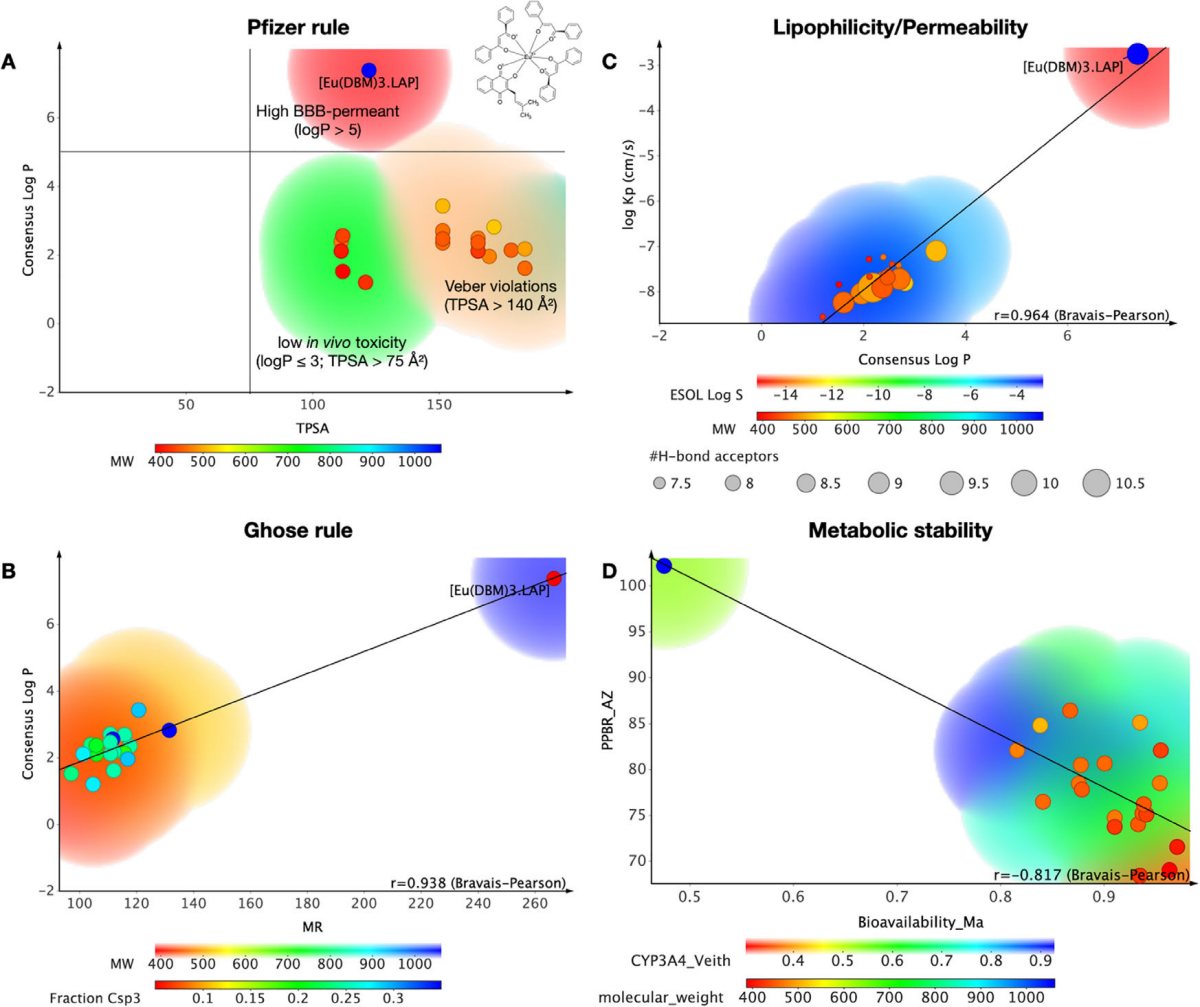
In silico analysis of the pharmacokinetic properties of the [Eu(dbm)_3_.LAP] complex, compared to other TRPA1 channel modulators present in the ChEMBL database. (A) Relationship between lipophilicity (logP) and polarity (TPSA), according to Pfizer's rule, indicating regions associated with high blood‐brain permeability (logP > 5) and low in vivo toxicity (logP ≤ 3; TPSA > 75 Å^2^). (B) Evaluation according to Ghose's rule, correlating logP with molar refractivity (MR). (C) Correlation between lipophilicity and permeability (logKp), showing a strong association (*r* = 0.964). (D) Inverse relationship between predicted metabolic stability (PPB) and bioavailability (*r* = −0.817). The color scales represent molecular weight (MW), solubility (ESOL log S), or affinity predicted by CYP3A4, while the size of the circles indicates the number of hydrogen bond acceptors.

When comparing [Eu(dbm)_3_.LAP] with compounds active against TRPA1 deposited in the ChEMBL database, it was observed that the organometallic complex resides in a physicochemical space formed by compounds that are highly permeable in the blood‐brain barrier (BBB) (red color spectra), according to the classification system of Pfizer, Inc. (Figure 8A), especially due to high lipophilicity (logP > 5), in addition to being more polar than drugs that showed toxicity in vivo (TPSA > 75 Å^2^) [40]. By applying the physicochemical properties to Ghose's rule (Figure 8B), it can be observed that the compound may have a higher molecular weight than conventional drugs (MW > 480 g/mol). In addition, MR > 130 suggests that dispersion forces promote the predominance of weak van der Waals interactions, indicating that the compound has a strong affinity for hydrophobic environments (Figure 8B) [41]. This attribute is strongly associated with the lipophilic profile observed by the calculated logP in the order of 7.38.

The regression of lipophilicity descriptors reveals a strong correlation between logP and logKp, with an *r* = 0.964 by the Bravais–Pearson matrix (Figure 8C). The results showed that [Eu(dbm)_3_.LAP] can be much more lipophilic (logP > 6) than other TRPA1 modulators, showing greater distance from the threshold of hydrophilic compounds (blue color spectra), which makes it much more permeable in cell membranes and biological tissues, with logKp calculated in the order of −2.75 cm/s, suggesting permeability kinetics in the order of 10^−3^ cm/s, a range associated with highly permeable compounds in cell lipid bilayers [42, 43, 44]. On the other hand, it is worth noting that the compound can bind strongly to plasma proteins, with a predicted PPB rate of ∼100% (Figure 8D), while TRPA1 modulating compounds from the ChEMBL database showed low affinity for serum proteins and therefore showed a larger volume of systemic distribution, indicating that [Eu(dbm)_3_.LAP] tends to distribute widely and diffuse into biological tissues [45]. The test showed an inversely proportional linear correlation between PPB and oral bioavailability fraction, with *r* = −0.817 according to the Bravais–Pearson matrix (Figure 8D), indicating that the organometallic complex basically follows the physicochemical trends that affect its pharmacokinetic properties. The selection of metal is associated with elevated lipophilicity, diminished renal excretion, and purported selective accumulation in tissues subject to permeability effects, thereby forming drugs with a prolonged plasma half‐life [46].

In addition, the compound showed a probability of around 0.53 of being a substrate of the major isoform in the human liver microsome (HLM) system, CYP3A4, indicating that it is a compound that can be biotransformed in first‐pass metabolism, which directly affects the oral bioavailability of the compound, that is, the molecular fraction that is absorbed and reaches the bloodstream to be distributed for therapeutic effect (Figure 8D) [45, 47].

**FIGURE 9:**
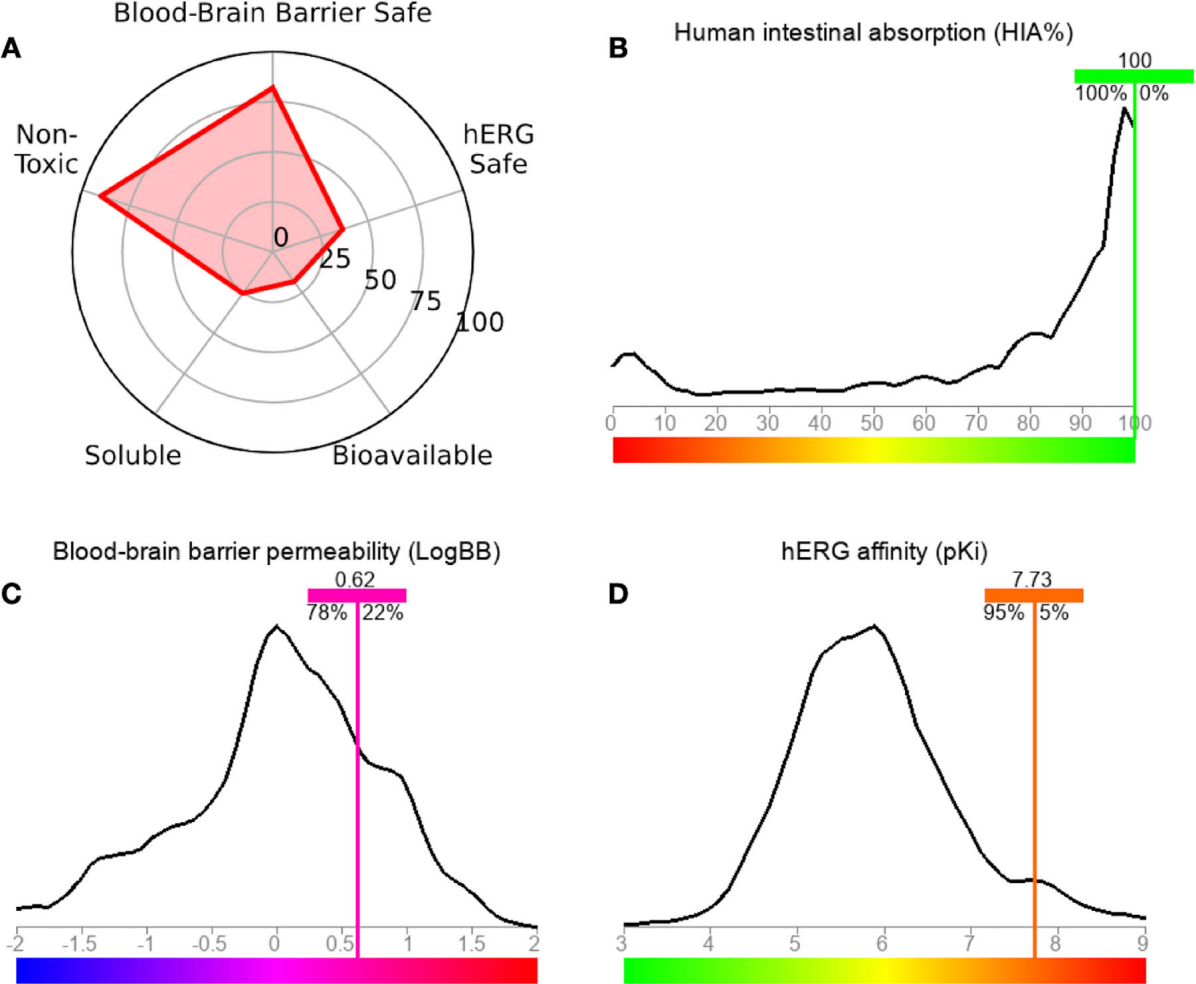
Predictions of pharmacokinetic and safety properties of the [Eu(dbm)_3_.LAP] complex. (A) Radar profile showing predictions of safety and pharmacokinetic performance, including toxicity, solubility, bioavailability, cardiac safety (hERG Safe), and blood‐brain barrier permeability (BBB Safe). (B) Predicted human intestinal absorption (HIA%), indicating high absorption (≈100%). (C) Permeability across the blood‐brain barrier (LogBB = 0.62), suggesting potential penetration into the central nervous system. (D) Predicted affinity for the hERG channel (pKi = 7.73), within the range considered safe for cardiac risk.

In general, a pharmacokinetic spectrum based on greater CNS safety due to BBB permeability and low oral bioavailability is predicted (Figure 9A). In the analysis of lipophilicity descriptors, it was possible to observe that the compound may be highly permeable in biological membranes, indicating that the bioavailable molecular fraction, although limited, readily penetrates the BBB. In the ADMET predictive test, an HIA rate statistically close to 100% was predicted (Figure 9B), as well as a BBB permeability coefficient (logBB) in the order of 0.62, showing a 78% similarity of data with molecular fragments that increase the BBB permeability of several drugs (Figure 9C) [48, 49]. In addition, the compound showed low safety probability in the hERG inhibition cardiotoxicity model (∼30%), with a predicted pKi of 7.73 indicating high affinity for hERG channels, showing at least 95% data similarity with molecular fragments contributing to the model (Figure 9D) [50]. The findings of this study corroborate ADMET's prediction for BBB permeability, as the treatment resulted in a substantial and highly significant reduction in oxidative stress in the brain, thereby demonstrating a neuroprotective effect that is superior to that of ibuprofen.

However, the validation of the models may be constrained due to the variety of predictive algorithms and units of measurement, necessitating experimental validation to substantiate the findings.

## 3 Conclusions

Given the results, the metal complex [Eu(dbm)_3_.LAP], synthesized from Lapachol, showed promising pharmacological effects by inhibiting nociceptive behavior in the TRPA1 channel, as well as anti‐inflammatory effects in adult zebrafish, generating hepatoprotective and neuroprotective effects against reactive oxygen species generated by the inflammatory process in carrageenan‐induced abdominal edema. Docking and molecular dynamics results indicate that the [Eu(dbm)_3_.LAP] complex exhibits high stability in the TRPA1 channel cavity, with less structural fluctuation and maintenance of key interactions observed for the native ligand. Combined with favorable ADMET predictions, these findings suggest a pharmacokinetic profile based on BBB permeation and potential modulatory activity on TRPA1. It can be concluded that the [Eu(dbm)_3_.LAP] complex is a promising candidate for use as an antinociceptive and anti‐inflammatory drug. The efficacy of its action via TRPA1 neuromodulation was validated by behavioral tests and refined by high‐precision molecular modeling. The favorable ADMET profile, including high intestinal absorption (∼100%) and dynamic stability, qualifies this organometallic compound for future clinical studies. It is imperative to acknowledge that experimental tests with the specific cell line (BBB‐like) are necessary to corroborate the pharmacokinetic profile of the complex. However, the present study does not encompass these tests.

## 4 Experimental Section

### 4.1 Synthesis of the Complex [Eu(dbm)_3_.LAP]

**FIGURE 10:**
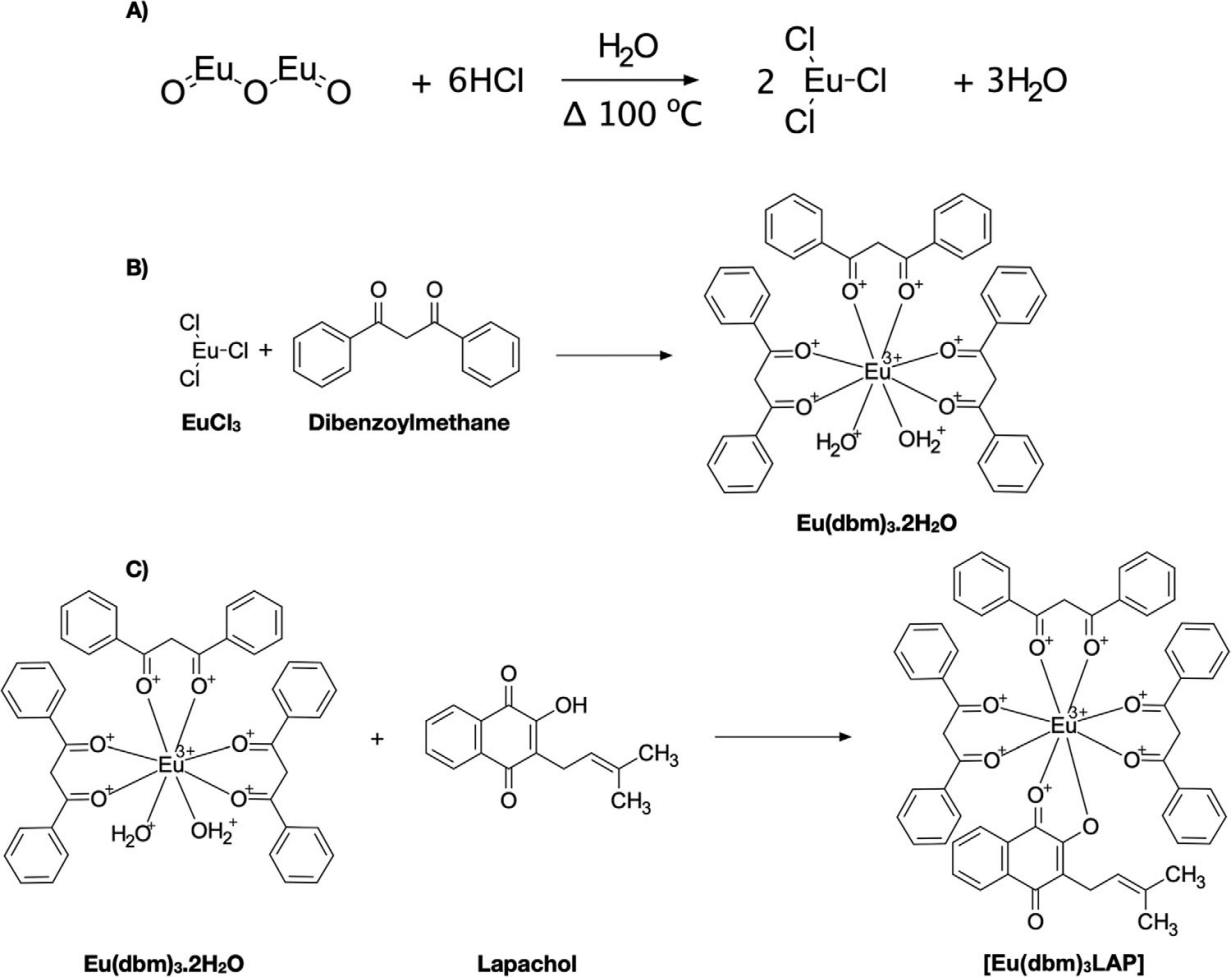
(A) Synthesis route of EuCl_3_.6H_2_O; (B) synthesis route of [Eu(DBM)3.(H2O)2]; (C) new complex synthesis route of [Eu(dbm)_3_.LAP]. Adapted from De Menezes et al. [51].

The synthesis of europium chloride was initiated with the precise measurement and subsequent dissolution of 1 g of Eu^3+^ oxide in 200 mL of distilled water within a 400 mL beaker. The system was subsequently placed on a magnetic stirrer and heated to a temperature of 100°C on a hot plate/stirrer. Subsequently, 2 mL of hydrochloric acid were added to the system in a gradual manner, continuing until only a negligible amount of oxide remained at the base of the beaker. The pH of the system was ascertained using indicator strips, and when it was determined to be between 5 and 6, the stirring and heating were halted, and the mixture underwent simple filtration. The clear liquid phase was collected and subjected to heating to evaporate the water until a white solid was obtained (Figure 10A) [51].

β‐Diketone, dibenzoylmethane (0.03 mol), was dissolved in 75 mL of 95% ethanol. Then, 30 mL (0.03 mol) of 1 M ammonium hydroxide was added to the alcoholic solution until the solution reached a pH of 7.0. A quantity of 0.01 mmol of EuCl3.6H_2_O, previously dissolved in 120 mL of distilled water, was added to the solution. The mixture was then subjected to magnetic stirring for approximately 2 h, until an oil became visible, which subsequently solidified. The solid was subjected to filtration, followed by a thorough wash with distilled water. Subsequently, it underwent a drying process in an oven. The compound was recrystallized with acetone and subsequently dried, washed with pentane, dried again, and stored under vacuum (Figure 10B) [51].

A quantity of 0.2 mmol of [Eu(dbm)_3_(H_2_O)_2_] was dissolved in approximately 30 mL of ethanol, along with 0.4 mmol of the ligand, LAP. Following the dissolution of the salt and ligand, the mixture was subjected to stirring for a period of approximately 2 h. The suspension was stored for a period of 10 days. Subsequently, the superior portion of the sample was extracted, and the crystals were methodically arranged into a clearly delineated configuration. The compound was washed using the same solvent that was used as the reaction medium (Figure 10C) [51].

### 4.2 Zebrafish

Zebrafish (*Danio rerio*), wild‐caught (90 to 120 days old; 0.4 ± 0.1 g, 3.5 ± 0.5 cm), both sexes, were obtained from a specialized company (Eusébio, CE). The animals were kept in 10 L aquariums (30 × 15 × 20 cm) (*n* = 3/L), with a circadian cycle of 10–14 h (light/dark), in dechlorinated water (ProtecPlus) and an air pump with submerged filters, at a temperature of 25°C and pH 7.0. The fish were fed ad libitum and Artemia salina 24 h before the experiments. Before drug administration, the animals were anesthetized in ice water, and after the experiments, they were sacrificed by immersion in ice water (2°C and 4°C) for 1 min until loss of opercular movements. The study was approved by the Animal Ethics Committee of the State University of Ceará (NUP 31032.000339/2025‐29) and was in accordance with the Ethical Principles of Animal Experimentation.

### 4.3 Behavioral Antinociceptive Activity

On the day of the experiments, fish were transferred to a damp sponge to receive oral (p.o.) treatments, which consisted of 20 µL doses of the metal complex sample [Eu(dbm)_3_.LAP] sample doses, or dimethyl sulfoxide (DMSO 3.0%) were applied orally (p.o.), formalin (TRPA1 channel agonist) was administered intramuscularly (im) in the tail, and camphor (TRPA1 channel antagonist) and/or morphine were applied intraperitoneally (ip). Subsequently, the animals were transferred to a glass beaker (250 mL) containing aquarium water to rest. Insulin syringes (0.5 mL; UltraFineVR BD) with a 30G needle were used for ip and im treatments, and applications were performed with a 20 µL variable automatic pipette [12].

### 4.4 Antinociceptive Mechanisms of Action via the TRPA1 Receptor

The fish (*n* = 6/group) were pretreated with camphor (30.4 mg/kg; 20 uL; ip), after 15 min the best effective dose of [Eu(dbm)_3_.LAP] (40 mg/kg; p.o.), and after 1 h, formalin was applied to the tail (0.1%; im) and open field analysis was performed. For the control groups, the following were used: control (DMSO –3%); or camphor (30.4 mg/kg; 20 uL; ip). After 1 h, formalin was applied, and open field analysis was performed. Untreated fish (Naive) (*n* = 6; group) were also included. The number of crossings in the open field was considered in both the neurogenic phase (0–5 min) and the inflammatory phase (15–30 min) [52].

### 4.5 Carrageenan‐Induced Anti‐Inflammatory Activity

Adult animals (*n* = 6/group) received [Eu(dbm)_3_.LAP], (40 mg/kg; p.o.), negative control (DMSO – 3.0%; 20 uL; p.o.) or ibuprofen (positive control, 100 mg/kg; 20 uL, ip) and after 40 min received an injection of 1.5% carrageenan (20 uL; ip). Each group had its animals weighed individually before treatment, and the animals in each group were weighed every hour after application, at intervals of 0–4 h, to assess the change in body weight caused by carrageenan‐induced edema [53]. The fish were sacrificed in an ice bath (4°C) after the experiment, and the brain and liver were removed for oxidative stress analysis.

### 4.6 Reactive Oxygen Species (ROS) Levels

After the inflammation test, the organs (brain and liver) were removed, macerated with Tris‐HCl‐EDTA, and centrifuged at 10 000 × *g* for 10 min at 4°C. After centrifugation, 50 µL of the supernatant was collected and mixed with 5 µL of 2′,7′‐dichlorofluorescein diacetate DCHF‐DA (5 mM). The fluorescence emission intensity was recorded at 520 nm (excitation at 480 nm) 60 min after the addition of DCHF‐DA.

### 4.7 Statistical Analysis

The results were expressed as the mean ± standard deviation of the mean of the in vivo tests (*n* = 6/group). The normality distribution and homogeneity of the data were analyzed using the Shapiro‐Wilk test. To compare behavioral parameters, differences between groups were subjected to one‐way ANOVA and Kruskal–Wallis, and in antagonist experiments to two‐way ANOVA, followed by Tukey's test, using GraphPad Prism v. 8.0 software. The level of statistical significance was set at 5% (*p* < 0.05).

### 4.8 Molecular Docking Simulations

All simulations were performed using free codes for academic use on a 64‐bit operating system. The following codes were used: AutoDockTools [54], AutoDockVina [55], Avogadro (http://avogadro.cc/) [56], Discovery Studio Visualizer Viewer [57], MarvinSketch, version 19.8.0 (http://www.chemaxon.com) and PyMOL [58].

**FIGURE 11:**
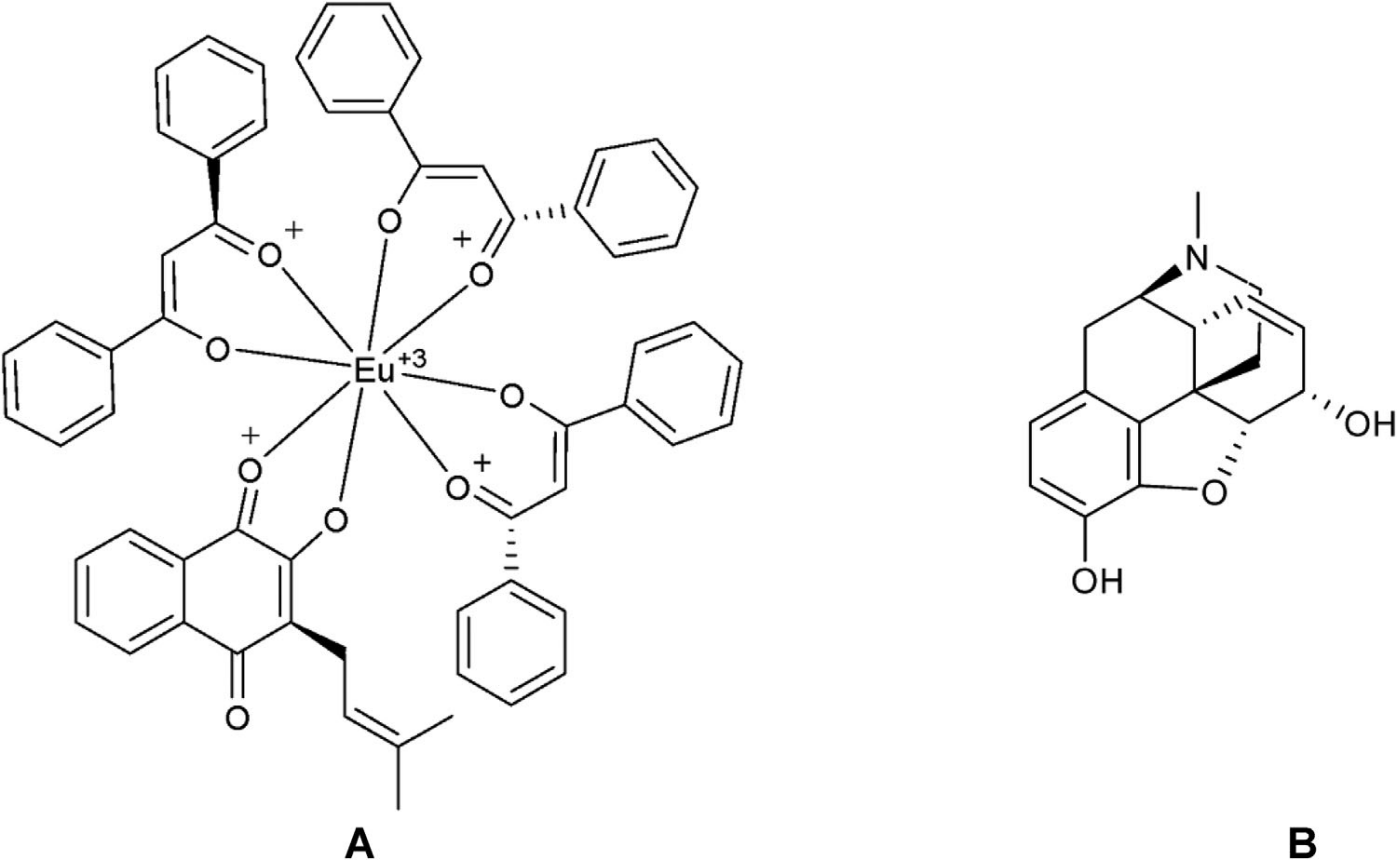
Generic 2D representation of the complexes generated for the project: (A) [Eu(dbm)_3_.LAP] and (B) morphine, created in ChemDraw.

The ligands used in the simulation: [Eu(dbm)_3_.LAP] and Morphine were created in MarvinSketch software (Figure 11); in the auto‐optimization settings, the MMFF94S force field was applied [59] to generate bioactive conformations by minimizing randomly generated conformers, using the Steepest Descent algorithm [60], with Avogadro software. All files with ligands were converted to corresponding formats (.mol2 and .pdbqt) with the addition of ionization and tautomeric states at pH 7.4 using OpenBabel software ver. 3.0.0 [61].

The cryo‐EM structure of the TRPA1 channel (PDB ID: 6 × 2J), in complex with its ligand, was obtained from the Protein Data Bank (PDB) and used as a model for the in silico experiments [62]. The molecular docking process was conducted using the Lamarckian Genetic Algorithm, as implemented in the AutoDockVina software [55]. For the preparation of the macromolecule, standard cleaning and optimization criteria were followed: all crystallographic water molecules were removed; Gasteiger partial charges were assigned; and essential polar hydrogens were added [63]. The preparation of the protein and ligand was performed using the AutoDockTools package [54]. To determine the simulation space, the grid box was centered to encompass all protein chains. The grid box was centered on the coordinates of its native co‐crystallized ligand (morphine) for the *x*, *y*, and *z* axes, 161.862577, 155.050615, and 139.771167, respectively, and had grid size parameters of 35 Å (*x*), 35 Å (*y*), and 35 Å (*z*).

To obtain a larger data set, 50 independent simulations were performed for all simulations (docking and redocking), yielding 20 poses per simulation. The exhaustivity criterion equal to 64 was used to improve the partial refinement of individual coupling calculations [64]. The protein structure was kept rigid, while all bonds and torques of the ligands were adjusted to rotate [65]. To statistically validate the simulations, refitting procedures were performed and root mean square deviation (RMSD) values were evaluated using a selection parameter of Best Pose values less than 2.5 Å [66]. To evaluate the strength of the hydrogen bond, the distance values between the donor and receptor atoms were used, with interactions between 2.5 and 3.1 Å classified as strong, those between 3.1 and 3.55 Å as medium, and those with a distance greater than 3.55 Å as weak [67]. The redocking technique was performed on the co‐crystallized ligands of each protein to validate the simulations, and the ligands were subjected to the same simulation criteria and conditions.

### 4.9 Molecular Dynamics Simulations

In order to conduct the molecular dynamics (MD) study, the atomic positions of the receptor‐ligand complex had to be obtained after carrying out molecular docking using AutoDockVina software, along with the topology files (PSF extension). MD simulations were then performed using the NAMD program [31]. The best conformations obtained in molecular docking were solvated in water using the TIP3P model [65] and the CHARMM36 force field [68]. System preparation was performed in two stages. First, the ligands were parameterized using the Charmm‐Gui server (https://www.charmm‐gui.org/) and then submitted to the CGenFF server to identify the CHARMM36 parameters [69]. In the second stage, the protein was prepared using the NAMD program. One Na^+^ ion per ligand was added to neutralize the total charge of the system, together with Cl^−^ ions. The system was then subjected to energy minimization using the steepest descent method. The system was then subjected to NVT and NPT equilibrations under the conditions described by Langevin [70]. Production simulations to study the system were performed for 100 ns. Morphine was used as a standard reference drug to analyze the interactions between the ligand and the protein.

The quality of the structures obtained in MD was evaluated using the following parameters with NAMD: potential energy (kcal/mol) [71]; protein‐ligand interaction energy (kcal/mol); root mean square deviation (RMSD, Å) of the protein, ligands, and distances between them; root mean square fluctuation (RMSF, Å), minimum distances between proteins and ligands observed in MD [72]. Hydrogen bonds were evaluated with visual molecular dynamics (VMD) [73]. The graphs will be generated using the Qtrace program [74].

### 4.10 MM/GBSA Calculations

The molecular mechanics energies combined with generalized continuous solvation of Born and MM/GBSA surface area were calculated by MolAICal [75] based on the MD .log file from NAMD software [31]. MM/GBSA is estimated by Equations (1–3):

(1) $$\Delta G_{bind}=\Delta H\;-\;T\Delta S\;\approx \;\Delta E_{MM}+\Delta G_{sol}\;-\;T\Delta S$$

(2)

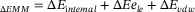



(3)

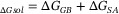

where Δ*E*_MM_, Δ*G*_sol_, and *T*Δ*S* represent the MM energy of the gas phase, solvation free energy (sum of the polar contribution Δ*G*_GB_ and nonpolar contribution Δ*G*_SA_), and conformational entropy, respectively. Δ*E*_MM_ contains van der Waals energy Δ*E*_vdw_, electrostatic energy Δ*E*_ele_, and Δ*E*_internal_ energy from bonding, angle, and dihedral energies. If there are no binding‐induced structural changes in the MD simulations, the entropy calculation can be omitted.

### 4.11 Multivariate ADMET Descriptors

To investigate the properties of drug metabolism and pharmacokinetics (DMPK), a multiparametric optimization (MPO) approach was chosen [76]. The two‐dimensional structure of [Eu(dbm)_3_.LAP] was plotted and converted into Simplified Molecular‐Input Line‐Entry System (SMILES) notation using MarvinSketch, version 24.1.0, Chemaxon (https://chemaxon.com/marvin), and subjected to a prediction of pharmacokinetic descriptors using the SwissADME (http://www.swissadme.ch/), ADMETlab 3.0 (https://admetlab3.scbdd.com/), pkCSM (https://biosig.lab.uq.edu.au/pkcsm/), and ADMET—LMC (https://qsar.chem.msu.ru/admet/) tools. With the results, a similarity test was performed with antibiotics deposited in the ChEMBL database (CHEMBL5737489) [77]. using the chemical intelligence (CI) software DataWarrior, version 06.01.00 (built Jan. 24, 2024) OpenChemLib (https://openmolecules.org/datawarrior/) [78, 79].

The physicochemical properties of lipophilicity (logP), molecular weight (MW), hydrogen bond donors (HBD), hydrogen bond acceptors (HBA), topological polar surface area (TPSA), fraction of sp^3^ hybridized carbons (Csp^3^) and molar refractivity (MR) were calculated from the structures plotted and converted into SMILES. These were applied to the drug‐likeness criteria to evaluate the potential of [Eu(dbm)_3_.LAP] as an oral drug using the SwissADME server (https://admetlab3.scbdd.com/). The drug‐likeness criteria used were the Pfizer rule for compounds with logP ≤ 3 and TPSA > 75 Å^2^ [40], and the Ghose rule for thresholds of MW ≤ 480 g/mol, logP ≤ 5.6, MR ≤ 130 and ≤ 70 atoms [41].

Pharmacokinetic properties include descriptors of permeability coefficient (logKp), human intestinal absorption (HIA), permeability in the blood‐brain barrier (BBB), plasma protein binding (PPB), oral bioavailability fraction, stability in the human liver microsome system (HLM) as a substrate of cytochrome P450 (CYP450) isoforms, and cardiotoxicity through inhibition of human Ether‐a‐go‐go‐Related Gene channels (hERG) [36, 80].

## Author Contributions

**Emanuela de Lima Rebouças Borges**: Conceptualization, writing – original draft preparation. **Antonio Wlisses da Silva**: Conceptualization, writing – original draft preparation. Erick Patrick Alves Moreira: Conceptualization, writing – original draft preparation. **Matheus Nunes da Rocha**: Conceptualization, writing – original draft preparation. **Hélcio Silva dos Santos**: supervision, Funding acquisition. **Jorge Fernando Silva de Menezes**: supervision, reviewing and editing of the manuscript. **Aluísio Marques da Fonseca**: supervision, reviewing and editing of the manuscript. **Maria Izabel Florindo Guedes**: supervision, reviewing and editing of the manuscript. **Emmanuel Silva Marinho**: supervision, Funding acquisition, reviewing and editing of the manuscript. all authors read and agreed to the final version of the manuscript.

## Conflicts of Interest

The authors declare no conflicts of interest.

## Data Availability

The data that support the findings of this study are available from the corresponding author upon reasonable request.
